# A new point cloud processing method unveiled hidden coastal boulders from deep vegetation

**DOI:** 10.1038/s41598-023-37985-2

**Published:** 2023-07-05

**Authors:** Koki Nakata, Hideaki Yanagisawa, Kazuhisa Goto

**Affiliations:** 1 Department of Earth and Planetary Science, The University of Tokyo, 7-3-1 Hongo, Bunkyo-ku, Tokyo, 113-0033 Japan; 2 Department of Regional Design, Faculty of Liberal Art, Tohoku Gakuin University, 2-1-1 Tenjinzawa, Izumi-ku, Sendai, Miyagi 981-3193 Japan

**Keywords:** Natural hazards, Ocean sciences

## Abstract

Huge coastal boulders are useful to reconstruct the size of past extreme waves such as those associated with tsunamis and storms using inverse-type or forward-type boulder transport models. These models fundamentally require the precise shape of boulders. Traditionally, they have often been assumed to be rectangular or ellipsoidal with three axes measured in the field. However, if the boulder’s shape is complex, this method is unable to represent the actual shape accurately. Therefore, it prevents estimation of the tsunami or storm size reasonably using models. For this reason, boulders have recently been surveyed using 3D scanning techniques such as LiDAR. However, coastal boulders now on land in tropical and subtropical areas such as Japan and Tonga are often covered by deep vegetation, which makes 3D surveys difficult. This report presents new methods to ascertain boulder shapes when they are obscured by vegetation. First, using UAV-type and mobile-type LiDAR, we scanned well-known tsunami boulders in southwestern Japan that had been covered with deep vegetation. Then, we developed a new method to extract only boulders and filter out vegetation from a point cloud. Thereby, we created 3D models of the boulders. We improved the boulder transport model further to assume the 3D boulder model accurately. In addition to coastal boulders, this filtering method is expected to be useful for unveiling any object, such as an archaeological structure, that is hidden in deep vegetation.

## Introduction

Around the world, coastal boulders transported from the sea are reported in coastal zones that include reefs and rocky cliffs^1–4^. Although the origins of some boulders remain uncertain, they are highly likely to have been transported by extreme waves such as tsunamis or storm waves. Once their origins (i.e. tsunami vs. storm) are confirmed, they can be crucially important for revealing the size and timing of their responsible extreme wave events. Inverse or forward models of boulder transport are often used to infer the sizes of past extreme wave events based on boulder attributes and locations^5,6^. Inverse models can estimate the minimum wave velocity necessary to move a boulder, whereas forward models can solve boulder transportation simultaneously by calculating tsunami propagation^7^. Because these models are based on Morison’s equation, which requires information related to the shape and density of boulders, precise measurements of boulder shapes and volumes are crucially important^8,9^. However, because of their extraordinarily large sizes, accurate measurements of boulders are not straightforward.

Over the last three decades, the volumes of coastal boulders have typically been estimated by measuring three axes (a, b, and c-axes) of boulders in the field and by assuming a rectangular or ellipsoidal shape. In recent years, with the advancement of field measurement technology, many studies have used new 3D surveying techniques such as photogrammetry and Light Detection And Ranging (LiDAR) for more accurate shape measurement of coastal boulders^10–14^. These 3D surveying techniques make it possible to measure the shapes and volumes of coastal boulders more accurately than conventional manual surveying. Therefore, these techniques are expected to improve the assessment of the size of paleo-tsunamis and storms drastically^9,13^.

**Figure 1:**
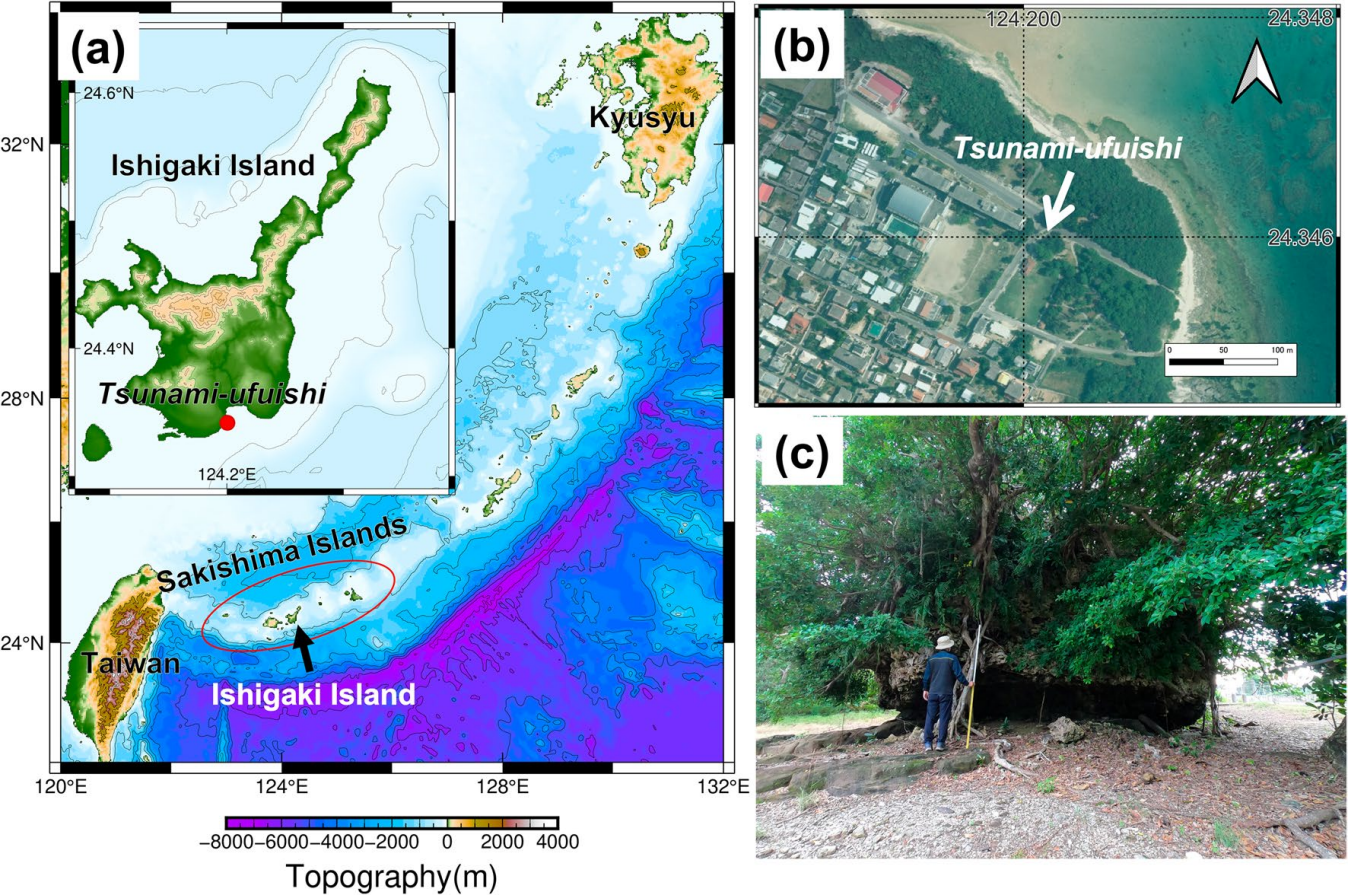
(**a**) Map showing the location of the “*Tsunami-ufuishi*” (TU boulder) on Ishigaki Island of the Sakishima Islands, Japan. (**b**) Aerial photograph of the TU boulder (white arrow) at Ohama, Ishigaki Island taken on 2015. The aerial photograph was provided by the Geospatial Information Authority of Japan. It is noteworthy that no rock surface is visible from the sky. (**c**) Field photograph of the TU boulder. The rock surface of the lower half of the boulder is visible, but the top surface is not visible from ground level by vegetation.

Photogrammetry and LiDAR are effective only if boulders are well exposed on the ground surface and if they are confirmed by a field survey to be unattached to bedrock. However, around the world, there are many boulders that are partially buried in a sand beach or covered by deep vegetation. In such situations, shape measurement is far more difficult. Measurement procedures are under development. For example, numerous coral boulders dot the reefs, beaches, and land areas of the Sakishima Islands of Japan^2,15^. Many of these boulders consist of single colonies of massive *Porites* sp., reef rocks that accumulated small corals such as table and massive corals, in addition to the Pleistocene Ryukyu limestone that makes up the islands with attached Holocene corals^15,16^. Because the Sakishima Islands are in the subtropical zone, these boulders tend to be covered with deep vegetation, especially those deposited on land. An example of them is the boulder called “*Tsunami-ufuishi*” in Ohama of the Ishigaki Island^2^ (Fig. 1), which we designate herein as the TU boulder. Because the TU boulder is huge, it is useful to constrain the fault parameters of past tsunamis^17^. Therefore, accurate measurement of this boulder shape is important to reconstruct the local size of past tsunamis. Nevertheless, measurement of this boulder manually in the field is extremely difficult because of its extraordinarily large size, together with the deep vegetation. Moreover, because the boulder is designated as a national monument, contactless measurement is necessary. Regarding 3D surveying, photogrammetry from aerial photography is inadequate to obtain point clouds of the boulder’s surface because of the deep vegetation covering the boulder’s top surface.

Instead, LiDAR techniques present the possibility of solving this problem. However, even if LiDAR is used for surveying, obtaining a point cloud of an irregularly shaped boulder surface beneath deep vegetation persists as a challenge. To do so, the vegetation must be removed from the point cloud data. Although filtering vegetation is commonly used to create a digital elevation model (DEM) from a digital surface model (DSM), the conventional method of filtering vegetation targets the 2.5-dimensional shape: a representation of the three-dimensional shape as seen from a single direction. The method is therefore unsuitable to filter out vegetation for three-dimensional (3D) shapes^18,19^. The vegetation covering the boulders, which are truly 3D shapes, cannot be filtered out using conventional filtering methods.

In this study, to resolve these difficulties of 3D and contactless measurement of huge and irregularly shaped boulders with deep vegetation, we first acquired point cloud data of the TU boulder using LiDAR. Subsequently, we developed a new method of point cloud analysis to extract the boulder shape, even in locations with deep vegetation.

## Geological setting

Ishigaki Island is surrounded by an approx. 1.5 km wide fringing reef with a few meters of water depth^2,20^. Numerous coral boulders are reported: sedimentological and numerical analyses have revealed that the boulders deposited along the coast and on land were deposited by large tsunami waves that struck the area in the past^2,21^. According to radiocarbon dating of the *Porites* tsunami boulders, large tsunamis repeatedly affected the Sakishima Islands with a 150–400 years recurrence interval^16^. The most recent large tsunami was the AD1771 Meiwa tsunami: its estimated maximum run-up height was about 30 m. In its wake, it left about 12,000 casualties^2^.

The TU boulder, located 100 m inland from the coast and at 10 m elevation^17^, is a reef rock with accumulated table corals, the species of which indicate that its original location was at the reef edge, approximately 700 m distant from its original position^22^. Fresh corals of this boulder are dated to 3480 yr BP at its base and to 1980 yr BP at its top^15^. Kawana and Nakata^15^ assumed that the boulder was deposited by an extremely large tsunami at about 2000 years ago. Hisamatsu et al.^17^ numerically estimated that two large tsunami events of equivalent size to the 1771 Meiwa tsunami might have been necessary to leave this boulder at this position after it was moved from its possible original location.

The size of the boulder was measured manually as 13 m (long axis) × 12 m (short axis) × 7.5 m (height) by Kawana and Nakata^15^ and as 12.4 m (long axis) × 10.8 m (short axis) × 5.9 m (height) by Hisamatsu et al.^17^ As these measurements show, up to 1.6 m difference was found between measurements by surveyors, suggesting the difficulty of measuring the size of a very large boulder. Currently, this boulder is densely wooded from the top, with trees growing up to 15 m above the ground. The bottom to the middle sides of the boulder surface are exposed, without coverage by vegetation. This boulder is designated as a national monument of Japan in 2013 so that contactless measurement is now required.

## Methods

### LiDAR surveying

LiDAR is a surveying system used to measure the distance to objects and to obtain point cloud data using a return pulse from a laser transmitter^23^. Unlike photogrammetry, LiDAR can be used to measure objects under the vegetation because the laser can reach the object surface through gaps in the vegetation^24^. Therefore, LiDAR is often used to generate DEM, not only in forested areas^25,26^ but also in archeological sites that are hidden by vegetation^27^. Therefore, LiDAR has some potential to measure a boulder covered by vegetation.

LiDAR has been developed to have three main types: Airborne Laser Scanning (ALS), Terrestrial Laser Scanning (TLS), and Mobile Laser Scanning (MLS)^28^. The first, ALS, can scan a wide area in a short time, but it has difficulty scanning the side to base of an object because it only scans from a top-to-down viewpoint^29,30^. The second, TLS, can provide highly accurate and detailed scanning, but when scanning an object such as a boulder using TLS, it is necessary to scan it from several directions to avoid leaving a non-scanned surface because these instruments are not moving. Furthermore, complex processes must be applied to combine these data scanned from several directions^31,32^. Moreover, when scanning the top surface of the object, TLS must be installed higher than the object's height. Depending on the height of the target objects and the surrounding environment, TLS might not be practical to scan the top surface^30,33^. The last of the methods, MLS, which is used with Simultaneous Localization and Mapping (SLAM) technique, can scan a wide area more easily than TLS and can cover most of the object in a single scan because it can scan while moving. Depending on the surrounding conditions and survey target, vehicle-mounted or backpack LiDAR is used as MLS^34^. However, similarly to TLS, it might be difficult to scan the top surface of an object by MLS higher than the height of the LiDAR position.

The TU boulder is about 7 m height. It is too tall to scan the top surface from ground level using either TLS or MLS. However, ALS can scan the top of the boulder, but it is difficult to scan the side of the boulder. Therefore, we used both ALS (UAV LiDAR) and MLS (Backpack LiDAR) to scan the boulder. Then we combined the point clouds acquired using each method to specifically ascertain the entire shape of the boulder.

The UAV LiDAR used for this study was Matrice300 RTK (DJI, China) with Zenmuse L1 as the LiDAR module. The measurement altitude was 50 m. We also used LiBackpack GC50 (GreenValley International, USA) as Backpack LiDAR, which has a SLAM system. The vertical field of view of LiBackpack GC50 is ± 15°. Both can provide high-precision positioning using GNSS. The LiDAR itself used in this study has accuracy of approx. 3 cm. The survey was administered in July 2022. We used DJI TERA software (DJI) for post-processing to the original data acquired using UAV LiDAR and LiFuser-BP software (GreenValley International) for post-processing of SLAM and GNSS to that by Backpack LiDAR. Strictly speaking, errors were found in location and elevation between the two sets of data. For this reason, the two data were aligned using Point Pairs Picking of CloudCompare software, while referring to artifacts near the TU boulder*.*

### Methods to make a 3D boulder model

**Figure 2:**
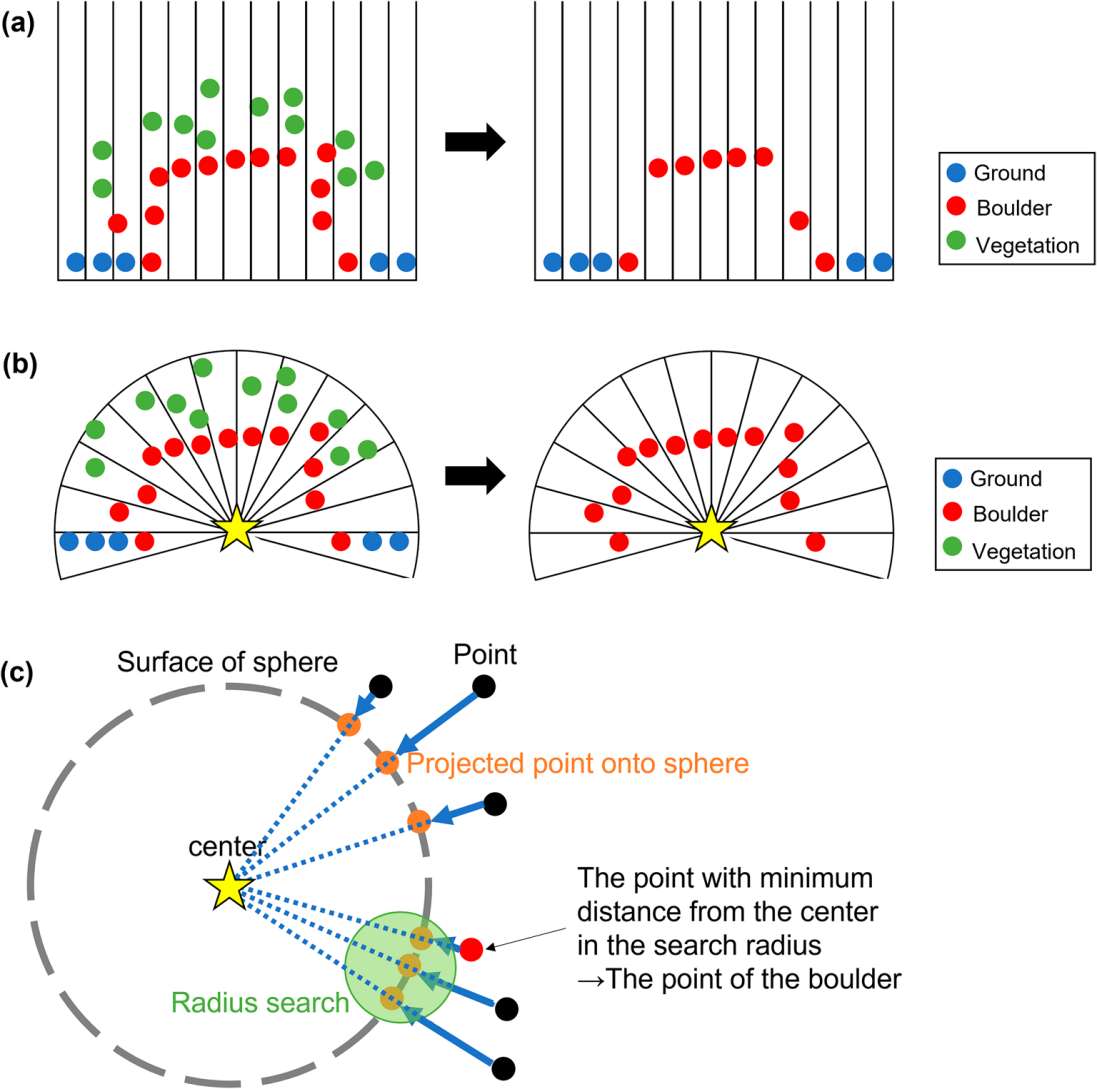
(**a**) Schematic figure of a traditional vegetation filtering method. Blue, red, and green circles respectively show ground, boulder, and vegetation points. The lowest point in each grid is extracted as non-vegetation, but it is unable to extract all points of the boulder appropriately. (**b**) Schematic figure of the new method of vegetation filter in this study. Points located at the shortest distance from a point inside the boulder (yellow star) are extracted as the boulder’s surface. (**c**) Schematic figure of the extraction method of surface point clouds of boulders using radius search.

After obtaining point cloud data, it was necessary to filter out the vegetation from the point cloud data and to extract only the points of the boulder to create a 3D model. In general, vegetation filtering, which is used to produce DEM from DSM, is based on the idea that the lowest height points in a certain width grid represent the ground surface^18^. However, although this method can extract 2.5D shapes such as topographic data, it is unable to extract 3D shapes such as boulders. Indeed, if this method were applied to the boulder, not only the vegetation on the boulder but also the boulder’s side surface would also be filtered out because they are not the lowest height points (Fig. 2a). Therefore, we developed a new method to filter vegetation on the boulder.

Our new idea was based on the fact that the innermost points represent the boulder's surface when looking outward from inside of the boulder (Fig. 2b). By our new approach, first, the viewpoint is set at the center of the boulder. The entire point cloud is projected onto the sphere centered on the viewpoint. For points projected onto the sphere, the points which have the minimum distance from the viewpoint originally among the neighboring points represent the boulder’s surface (Fig. 2c). Then, we extracted the points at the minimum distance within the neighboring points by a radius search using kd-tree^35^. In this process, if the shape of the boulders is complex, then portions of the boulder might be in the shadow of itself when viewed only from the center of the boulder: the points of the shadowed area of the boulder can then not be extracted. Therefore, to avoid producing a shadowed area, multiple viewpoints were set up in addition to the center of the boulder. Then, the processing above was performed at each viewpoint. All point clouds extracted by each viewpoint were combined.

Moreover, considering the accuracy of LiDAR itself, the extracted points were not used as the boulder surface points directly. A new point was set at the average coordinates of the points within a few centimeters from each extracted point. Then, these new points were adopted as the boulder surface points.

If the search radius is too large, then the extracted point cloud will include vegetation, whereas only small points of the boulder can be extracted if it is too small. Therefore, the search radius must be set appropriately. For this study, the search radius was set to 0.01 m. However, even if setting the appropriate value of the search radius, points other than the boulder might still remain. In this case, we cleaned the noise manually.

After extracting the point clouds of the boulder, a polygon mesh was created by Poisson Surface Reconstruction^36^ to determine the surface of the boulder. The maximum value of depth, which determines the resolution of the mesh, was set to 10. These processes were performed using Point Cloud Library^37^.

## Results

### The 3D model of the TU boulder

**Figure 3:**
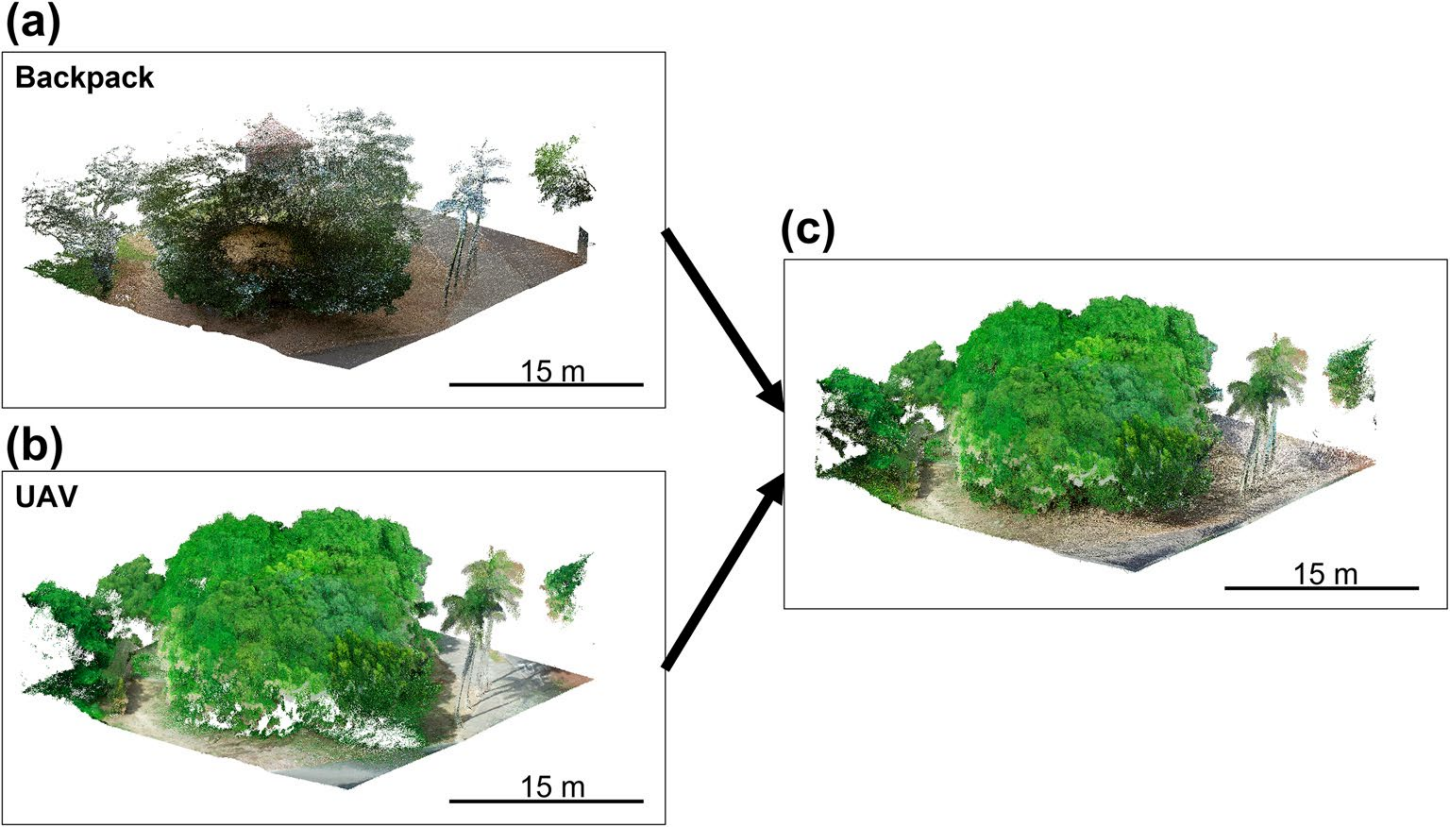
Point cloud data of the TU boulder obtained by (**a**) Backpack LiDAR, (**b**) UAV LiDAR, and (**c**) then integrated as the complete point cloud data.

**Figure 4:**
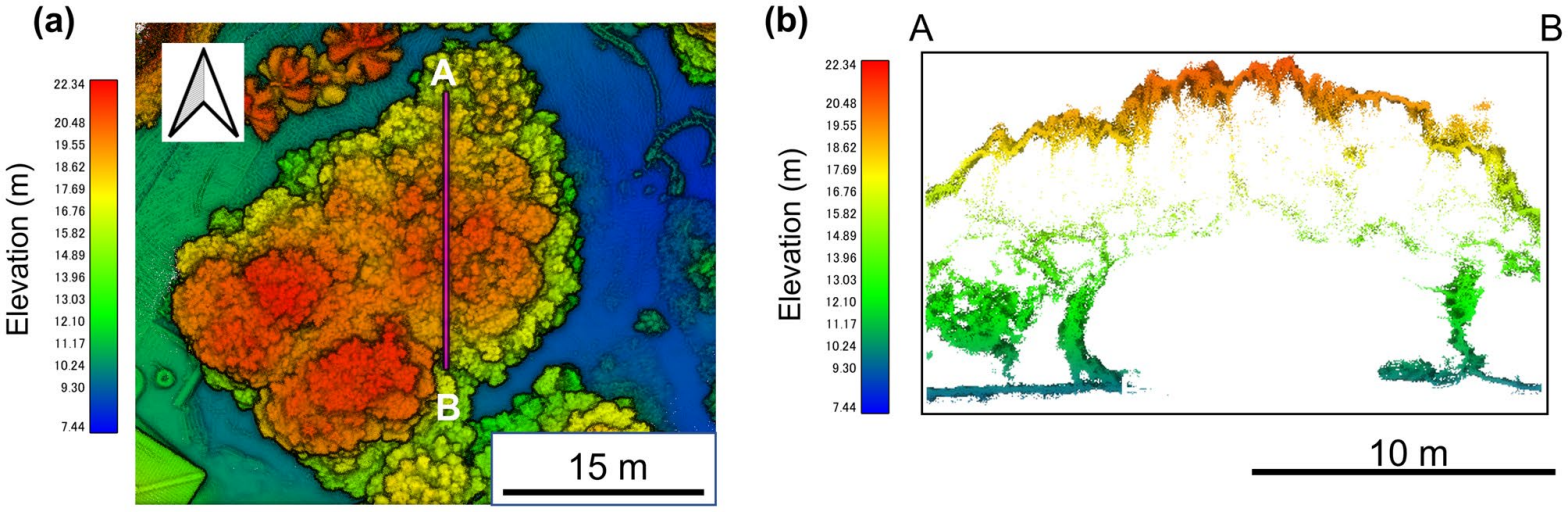
(**a**) Height map using point cloud around the TU boulder. (**b**) Cross-section of a point cloud of the TU boulder. A and B represent each end of the transect as depicted in Fig. 4a.

**Figure 5:**
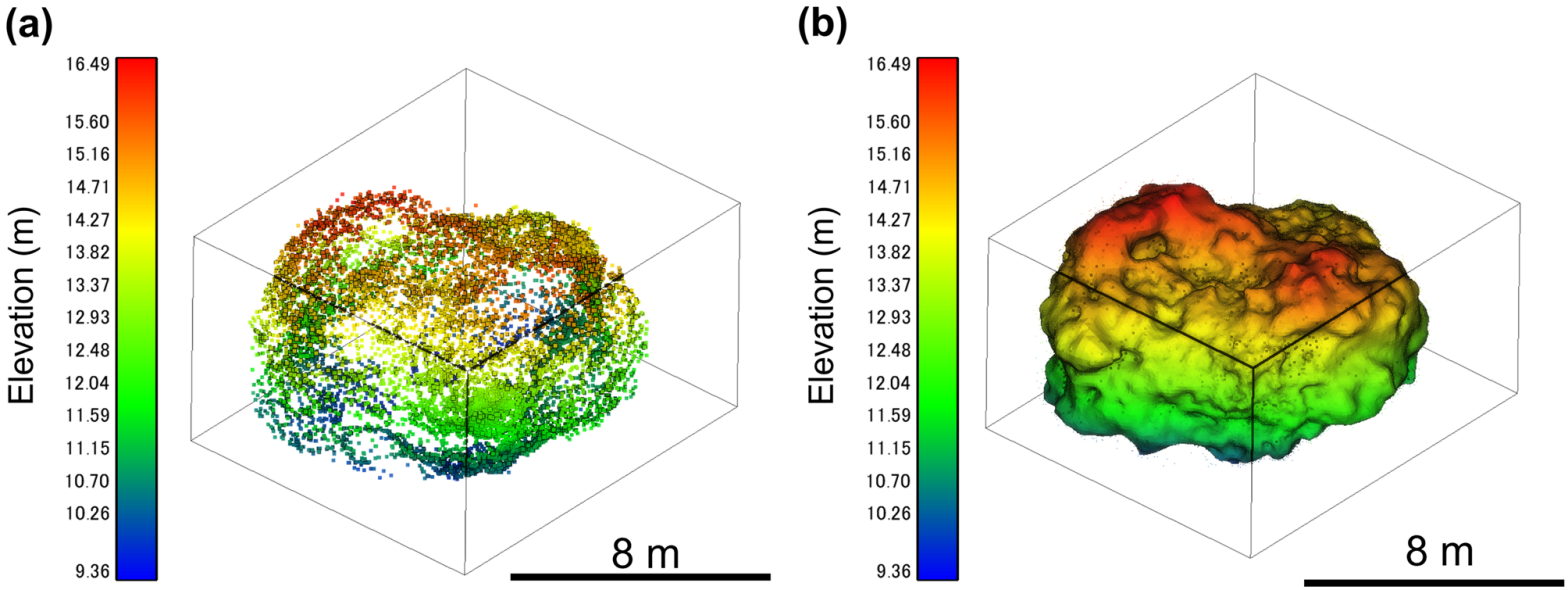
(**a**) Point cloud data of the TU boulder after removing vegetation. (**b**) 3D model of the TU boulder*.*

Although UAV LiDAR acquired the point cloud of the top of the TU boulder, it did not acquire that of its side and bottom. However, Backpack LiDAR obtained the point cloud data from the bottom to the side of the boulder, but not its top. Therefore, we integrated these data for mutual interpolation to make a complete point cloud dataset (Fig. 3). Looking at the cross-section of this integrated point cloud data, a blank shape, which is inside of the point cloud, represented the boulder (Fig. 4). The radar of LiDAR was reflected on the boulder’s surface, but it did not penetrate to its interior: consequently, the boulder is represented as a blank shape. In this case, the innermost points around the boulder outlined the boulder’s surface. From this point cloud data, 15,301 points were extracted as the boulder’s surface. Finally, a 3D model of the boulder without vegetation was obtained (Fig. 5). The volume of the TU boulder calculated from the 3D model was V = 384 m^3^. The three axis lengths of the 3D model would be 12.7 × 11.0 × 7.13 m if we were to fold this boulder into a rectangular box.

### Comparison of the 3D model and the actual shape

**Figure 6:**
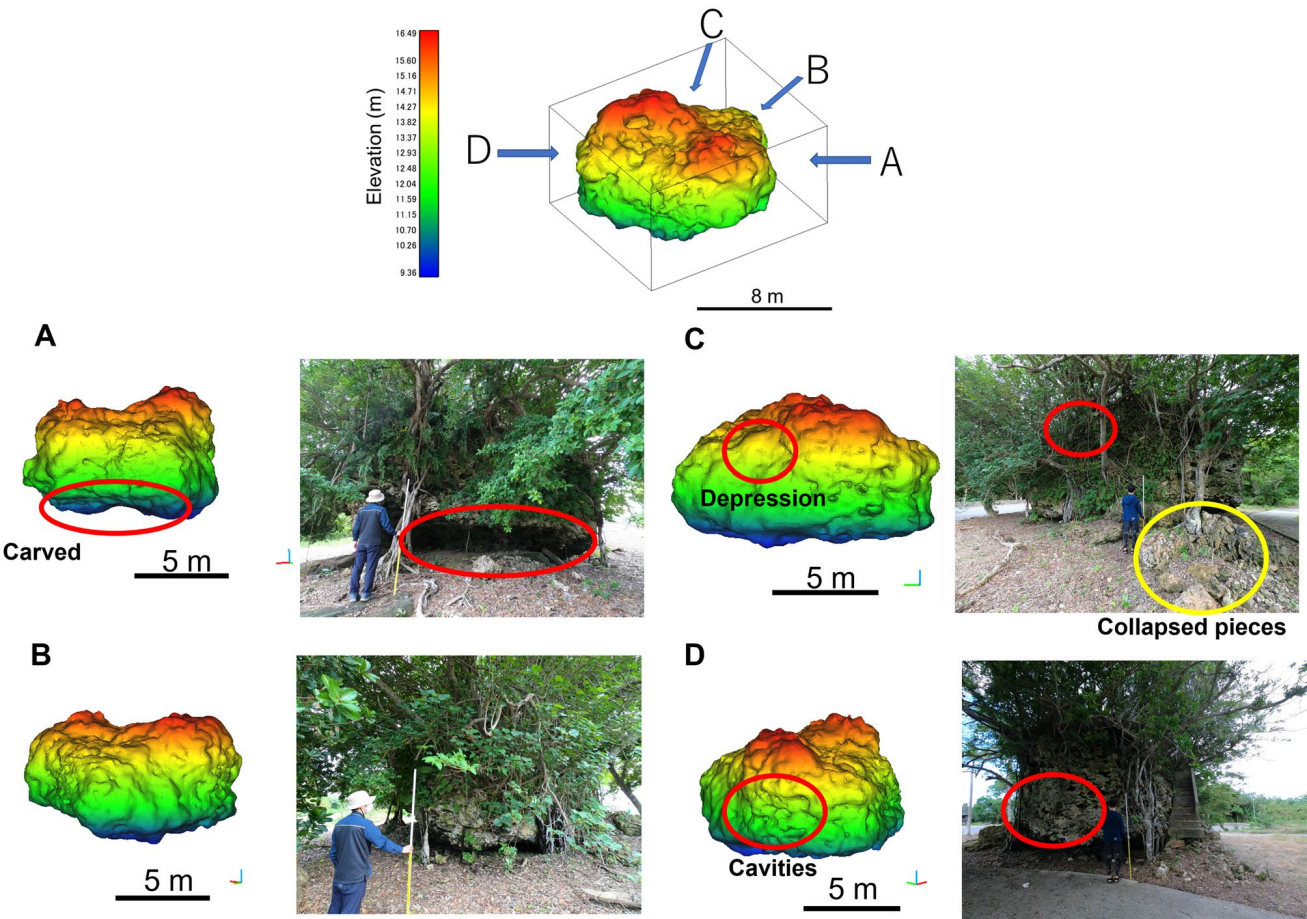
Comparison of the actual shape of the TU boulder to its 3D model created for this study.

We compared the actual shape of the boulder with that of the 3D model. As Fig. 6 shows, beneath the actual boulder is carved, looking from the A direction. From the B direction, the actual boulder was lower on the near side than the surrounding. From the C direction, the actual boulder has a depression at the top of the left side. The depression was presumed to result from the collapse of the boulder on field observation. The collapsed pieces spread to the ground around it. From direction D, the actual boulder surface had some large cavities. The 3D model well reproduced all these features from the A to D directions.

### Differences in point cloud processing results according to parameters

**Figure 7:**
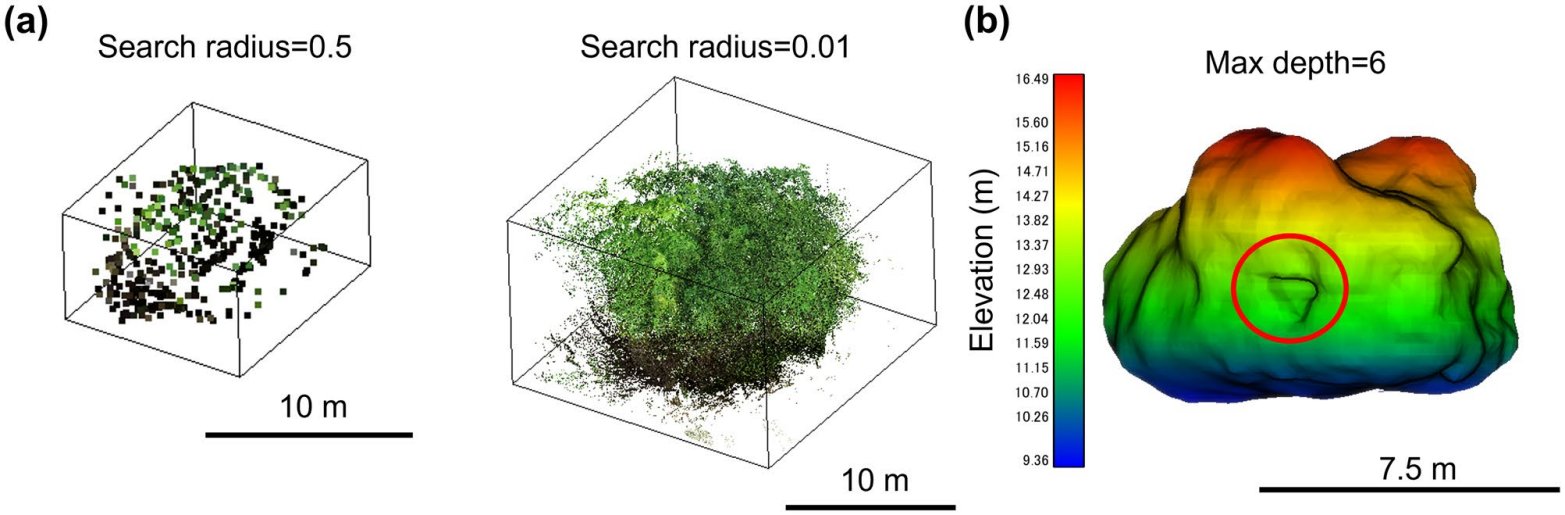
(**a**) Difference in the extracted point clouds by different search radii. Search radius = 0.5 (left) and Search radius = 0.01 (right). (**b**) The 3D model of the TU boulder when polygonized at Max depth = 6, viewed from the D direction in Fig. 6. The red circle represents a single hole, but originally there were some cavities on the surface.

We checked the differences in the processing results by the radius of the search circle and the depth of the Poisson Surface Reconstruction (PSR). If the radius of the search circle is too large (0.5 m), then a few points of the boulder can be extracted (number of extracted points = 471) (Fig. 7a). However, if the search radius is too small (0.01 m), then not only the points of the boulder but also those of the vegetation are extracted as well (Fig. 7a). In addition, when olygonising the points of the TU boulder with Max depth = 6 by PSR, the boulder surface is smoother than with Max depth = 10 (Fig. 5) and some large cavities which are visible from the D direction in Fig. 6 are reproduced as a single cavity (Fig. 7b).

## Discussion

### How accurate is the 3D model of the TU boulder?

In principle, because the accuracy of LiDAR itself is approximately 3 cm, this size of objects such as small branches and table corals should be reproduced. However, the accuracy would be lower than the ideal condition because many obstacles such as vegetation prevent one from obtaining sufficient points to make a 3D model with such accuracy. Furthermore, when the unorganized point cloud derived from LiDAR is polygonized, reconstruction of the precise surface from the point cloud is a difficult problem^38^. The 3D model created for this study at least reproduced the cavities of about 30 cm long on the boulder’s surface (Fig. 6). Presumably, it reproduced the actual boulder with this order of accuracy.

By the same method, we also measured one other coastal boulder on Ishigaki Island, which is completely covered by deep vegetation. Results showed that a 3D model of this boulder was reproduced successfully, although the number of the obtained point cloud is two orders fewer than that of the TU boulder (Supplementary information). Therefore, the method developed for this study is useful for the 3D surveying of any boulders covered by vegetation, but the number of obtained point cloud data depends on the rate of the boulder coverage by vegetation.

It is important to note that the parameters such as the radius search and the PSR should be adequately assumed to reproduce the densely vegetated boulders appropriately. If the radius is too small, then the points of the boulder might not be within the search radius. The points of vegetation might be extracted incorrectly as the boulder’s surface. However, if the radius is too large, then only a few points of the boulder are extracted. Therefore, the detailed shape of the boulder is not reproducible (Fig. 7).

Another important parameter is PSR. If the depth is set too deep, then the 3D model will be smoothed rough; small irregularities on the boulder surface are not reproducible (Fig. 7). By contrast, if it is too small, then the 3D model becomes more complex than the actual shape.

In this way, it is necessary to set appropriate parameters for vegetation filtering and polygonization. However, the appropriate parameters might vary depending on the boulder shape and the point cloud data. Therefore, the parameters should be ascertained through trial and error by comparison with the actual shape and 3D model carefully.

In this study, we developed a contactless method to extract the shape of vegetation-covered boulders from point cloud data obtained by LiDAR. However, accuracy of the shape may vary depending on vegetation parameters such as vegetation species, density, and height. Effect of vegetation can be represented by the shielding ratio. For example, it has been reported that the higher the shielding ratio by vegetation, the smaller the transmission rate of radar emitted from LiDAR: the transmission rate becomes around 20% when the shielding rate is above 70%^39^. Specifically for the LiDAR equipments that we used in this study, the effect of shielding by vegetation has also been preliminary evaluated: the ground surface of nearby mangrove forests cannot be adequately captured when shielding ratio exceeds about 85% (K. Kasai, personal communication). Although vegetation is different, this result could also be applied to our measurements. The issues of shielding by vegetation and transmittance are considered to vary depending on the performance of LiDAR equipment that one uses and thus individual evaluation of each LiDAR equipment is required.

### Updating the boulder transport model

The TU boulder has contributed to elucidation of the history of paleotsunamis that have struck Ishigaki Island^15,40^. Additionally, because of its extremely large size, it is useful to estimate the local size of paleotsunamis using the boulder transport model^17^. Indeed, Hisamatsu et al.^17^ evaluated the scale of the 1771 Meiwa tsunami and earlier tsunamis by calculating the TU boulder movement. They conducted manual surveys of the TU boulder on the field and reported that the length of its three axes as 12.4 × 10.8 × 5.9 m. From the 3D survey of this study, the three axes were measured as 12.7 × 11.0 × 7.13 m. Comparison of the two results shows the major and minor axes to be almost identical, whereas the height differed by 1.2 m. Two points might account for this difference. One is the difficulty of measuring the very large boulder manually. Second is that the top of the boulder is not visible from the ground because of the deep vegetation growing on the top of it. Therefore, manual surveying of very large boulders covered by vegetation is not straightforward. Creating a 3D model, as demonstrated in this study, is desirable.

Traditionally, the boulder shape was assumed as rectangular or ellipsoid in the forward and inverse boulder transport models, as Hisamatsu et al.^17^ did with the ellipsoidal assumption of the TU boulder. This is a simple and reasonable assumption, but this assumption sometimes leads to overestimation of the volume and weight depending on the boulder shape. For instance, the volume of the TU boulder with the assumption of ellipsoidal shape is calculated as 413 m^3^. Its weight is 535 tons based on its density (1.62 tons/m^3^) and porosity (20%)^17^. The volume of the 3D model in this study is 384 m^3^. Its weight is estimated as 498 tons based on the same assumptions of density and porosity. Therefore, both the volume and weight of the boulder is overestimated by 7% in the study reported by Hisamatsu et al.^17^.

**Figure 8:**
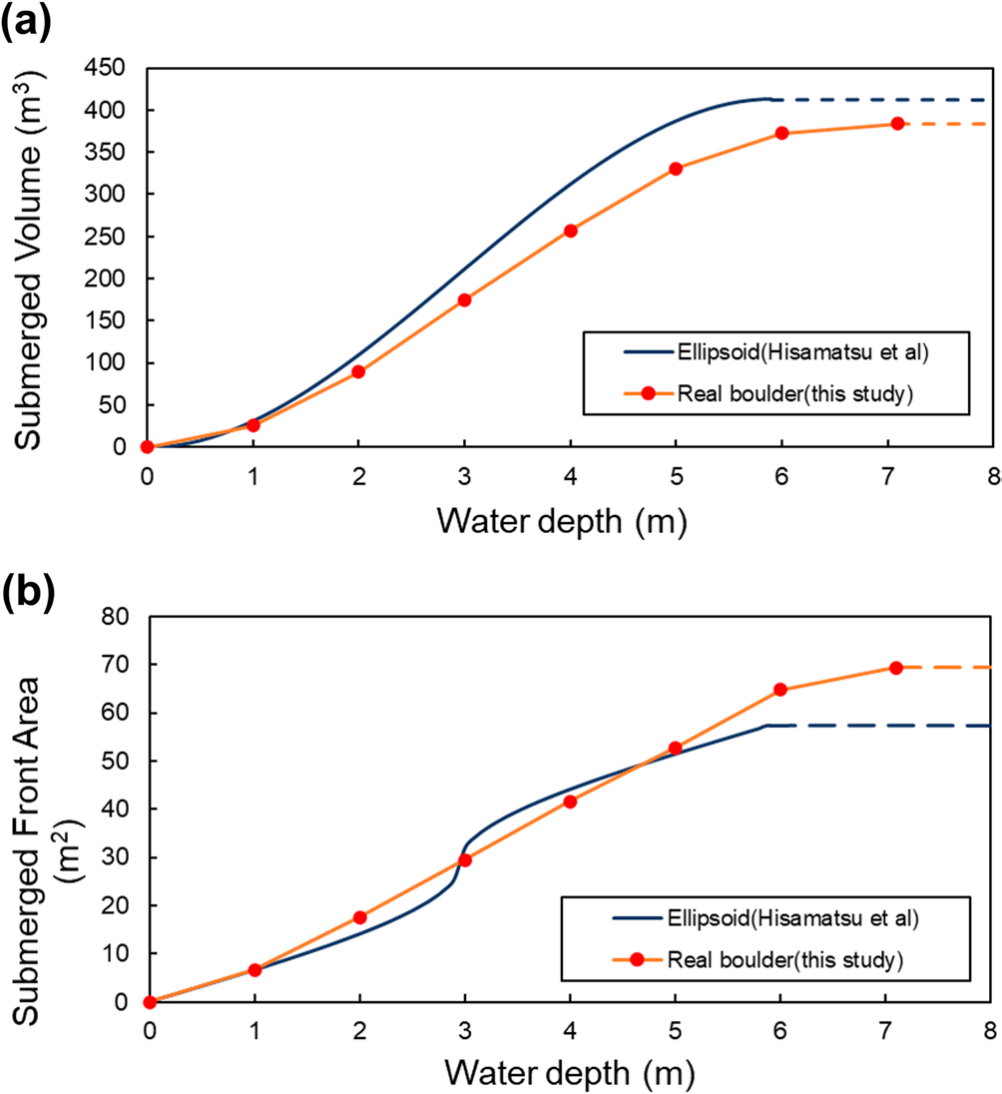
(**a**) Cumulative distribution of submerged volume with each height (= water depth) of the TU boulder. (**b**) Cumulative distribution of submerged frontal area parallel to the long axis with each water depth of the TU boulder. The dark blue line shows the ellipsoid case^17^; the red circle shows the value from the 3D model.

Both forward and inverse models are based on the classical Morison’s formula^7^. Because the volume and weight are important parameters for this formula, this overestimation prevents the accurate estimation of the tsunami size by the model^8^. Moreover, in the forward model proposed by Imamura et al.^5^ as used in Hisamatsu et al.^17^, not only the boulder weight, but also its submerged area and the volume of the boulder are crucially important to calculate the buoyancy and submerged frontal area parallel to the long axis and to calculate the drag force for each water depth adequately^5,41^. In Fig. 8, the submerged volume and frontal area parallel to the long axis of the 3D model are shown as a solid curve. Those of the ellipsoid assumed in Hisamatsu et al.^17^ are shown as a point. The maximum value is extended as a dotted line in the subsequent parts because the submerged volume and front area do not change with water depth beyond the boulder height. For the ellipsoid boulder assumed in Hisamatsu et al.^17^, the submerged volume is slightly underestimated at any water depth, with a maximum error of about 19% at water depth of 2 m. The submerged frontal area shows good agreement up to water depth of 5 m, but it is overestimated at greater water depths, with a maximum error of about 20% at water depths greater than 7.13 m.

Actually, the ellipsoidal assumption by Hisamatsu et al.^17^ showed good agreement with the 3D model within approximately 20% error at all depths, probably because the TU boulder originally resembled an ellipsoid. For that reason, the approximation to the ellipsoid worked well. However, more irregular shaped boulders are more difficult to measure by the manual survey. Therefore, a 3D survey is necessary to measure the shape precisely.

In addition, the forward boulder transport model proposed by Imamura et al.^5^ is designed for rectangular or ellipsoids. Therefore, it cannot use full information of 3D models directly. Therefore, the forward model should be updated to use the full information of the boulder’s 3D model. The better approach for the updating is to use the frontal area, volume, and weight of the boulder based on their cumulative distribution against the boulder’s height (Fig. 8), and then inserting the regression curves directly in the model. This method can be expected to be simple and easy. It is expected to be a powerful tool for representing the irregular shape of boulders in the model.

### Unveiling any object under the deep vegetation

The benefits of 3D surveying of the boulder are not limited to improvement of the boulder transport model. For example, the TU boulder is a Japanese national natural monument. Its conservation is therefore an important issue. If the shape can be measured non-destructively as in this method, then it is possible to ascertain the rock weathering process. This knowledge can be expected to facilitate the consideration of measures of the minimum necessary treatments. In addition, coastal boulders covered by vegetation have been reported worldwide, and particularly in the Philippines^42^ and Tonga^43^. Our method is expected to be useful for the 3D survey of these boulders.

Our method is applicable to any object under deep vegetation. As an example of the investigation of archaeological sites, the remains covered by deep vegetation have been reported worldwide, such as Angkor Wat and Maya. Ascertaining the shapes of such archeological structures non-destructively is extremely valuable. Several studies have been conducted using UAV LiDAR to survey archaeological sites in forested areas^27,44^. However, as this study demonstrates, point cloud data scanned from the sky might not be sufficient to ascertain the 3D shape of a complex object because the side and bottom shapes might not be reproduced well. Therefore, combinations of ALS and MLS (or TLS) are necessary. Once a complete set of point cloud data is obtained, the method proposed in this study can serve as an extremely powerful tool to extract the detailed shapes of such archeological structures from vegetation.

Another example of the application of this method is vegetation filtering on terrain with steep slopes, such as overhanging cliffs. Conventionally, methods such as taking the lowest points in a point cloud have been used for vegetation filtering^18^. However, such methods present difficulties: specifically, extracting steep slopes such as overhanging cliffs as a ground surface is complicated and onerous^18,19^. Although a few reports have described studies^19^ attempting to resolve these difficulties, no method for vegetation filtering on steep slopes has been established. Therefore, our method is one candidate for such method. It is possible to filter vegetation even on a steep slope, extracting the innermost points when viewed from the inside of the cliff to the outside. Therefore, our method has potential for use as a new vegetation filtering method for any complex topography.

## Supplementary Information

Supplementary Information.

## Data Availability

The datasets used and/or analyzed during the current study are available from the corresponding author on reasonable request.
