# Supplementary material for: A new point cloud processing method unveiled hidden coastal boulders from deep vegetation
Source: Sci Rep. 2023 Jul 5;13:10918. doi: 10.1038/s41598-023-37985-2 (PMC10322928; doi:10.1038/s41598-023-37985-2)
Supplement: Supplementary file 1 — Supplementary Information. [file 41598_2023_37985_MOESM1_ESM.docx]

**Supplementary information**

**Unveiling hidden coastal boulders from deep vegetation**

Koki Nakata^1*^, Hideaki Yanagisawa^2^, Kazuhisa Goto^1^

^1^ Department of Earth and Planetary Science, The University of Tokyo, 7-3-1 Hongo, Bunkyo-ku, Tokyo, 113-0033, Japan

^2^ Department of Regional Design, Faculty of Liberal Art, Tohoku Gakuin University, 2-1-1 Tenjinzawa, Izumi-ku, Sendai, Miyagi, 981-3193, Japan.

^*^Corresponding author: Koki Nakata (nakata-koki864@g.ecc.u-tokyo.ac.jp)

**Supplementary information**

In this study, we surveyed not only *“Tsunami-ufuishi”* (TU boulder) but also another coastal boulder called *“Amatariya-suuari”* (AS boulder) in Inoda, Ishigaki Island (Fig. S1). The AS boulder, which is composed of the Pleistocene Ryukyu limestone, is located approximately 200 m inland from the coast and at 6.7 m elevation^1^. Based on historical descriptions, the 1771 Meiwa tsunami transported the boulder from 320 m offshore from the coastline to the present position^2^. Today, trees are growing from the entire surface of the AS boulder, and the boulder is completely hidden by deep vegetation. Therefore, it is unable to see the boulder’s surface from any direction (Fig. S1).

In the case of the AS boulder, UAV LiDAR can scan not only the top surface but also the side of the boulder, so we used only point cloud data obtained by UAV LiDAR. Looking at a cross-sectional view of a certain section, the boulder appears to have a gap and is cracked (Fig. S2). However, we checked the boulder in the field, then we found that there is not only one boulder but also artificially piled stones next to it beneath the vegetation (Fig. S3). This indicates that it is important to observe the boulder in the field, rather than relying only on point cloud data or images.

In this study, we processed to extract only the points of the boulder, not including those of the piled stones (Fig. S3). The extracted point cloud of the boulder consists of 263 points (Fig. S4). Compared to the TU boulder, the number of points is fewer with two orders. This is because the entire area of the boulder is almost completely covered by vegetation. Therefore, the 3D model of the AS boulder can only reproduce the approximate shape of larger scale than that of the TU boulder. It should be noted that the detailed shape comparison between the 3D model and the actual boulder as was done for the TU boulder is difficult because the AS boulder is mostly invisible. The volume of the boulder calculated from the 3D model was V=130 m^3^, and the three axes were 9.7 m×8.2 m×3.75 m, assuming a rectangular shape in which the boulder completely fits.

**
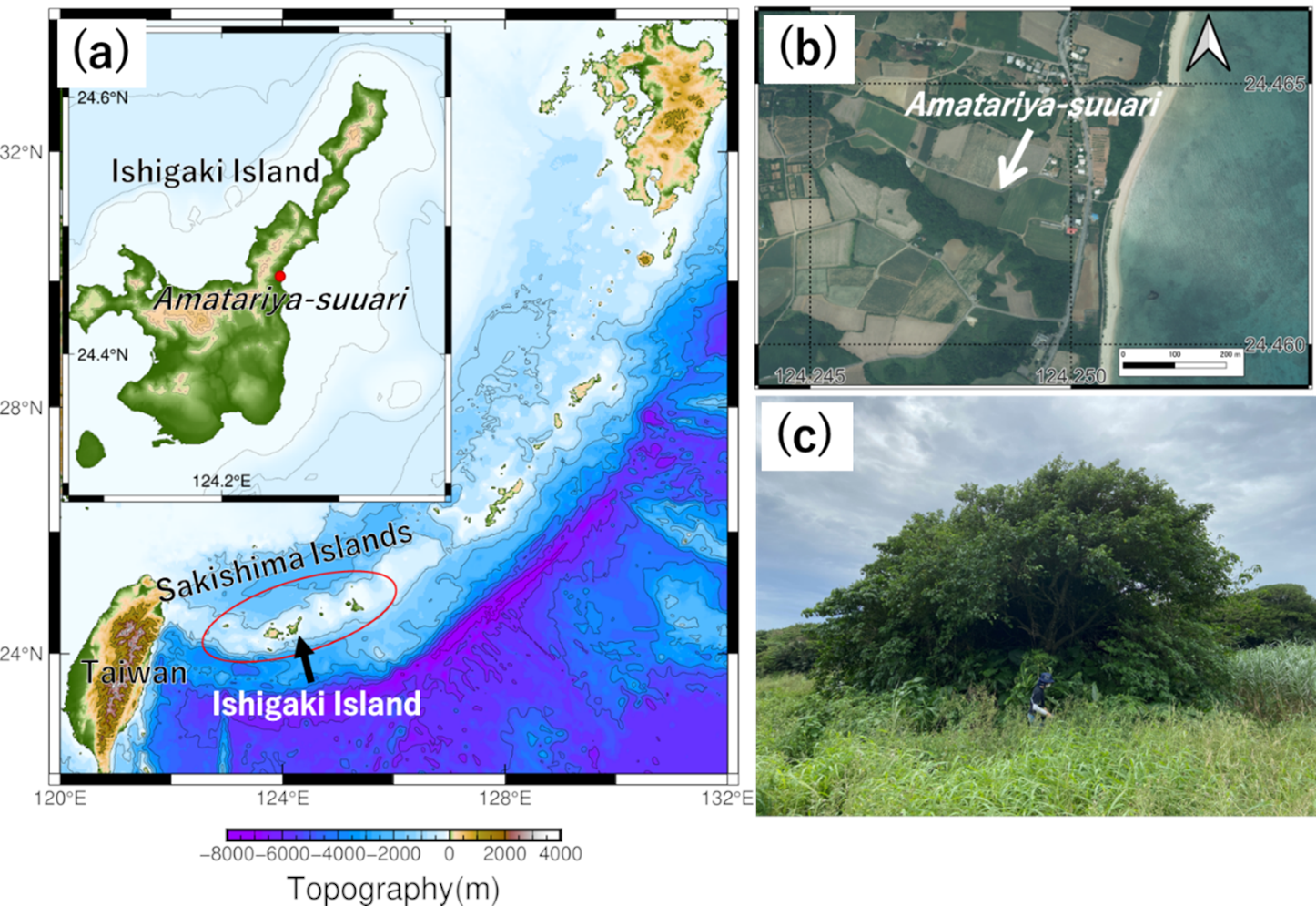
**

**Figure. S1**

(a) A map showing the location of the “*Amatariya-suuari*” (AS boulder) in Ishigaki Island of Sakishima Islands. (b) Aerial photograph of the AS boulder (white arrow) at Inoda, Ishigaki Island. The aerial photograph was taken on 2015 and provided by the Geospatial Information Authority of Japan. Note that no rock surface is visible from the sky. (c) Field photograph of the AS boulder. The boulder surface is hidden by deep vegetation and is invisible.


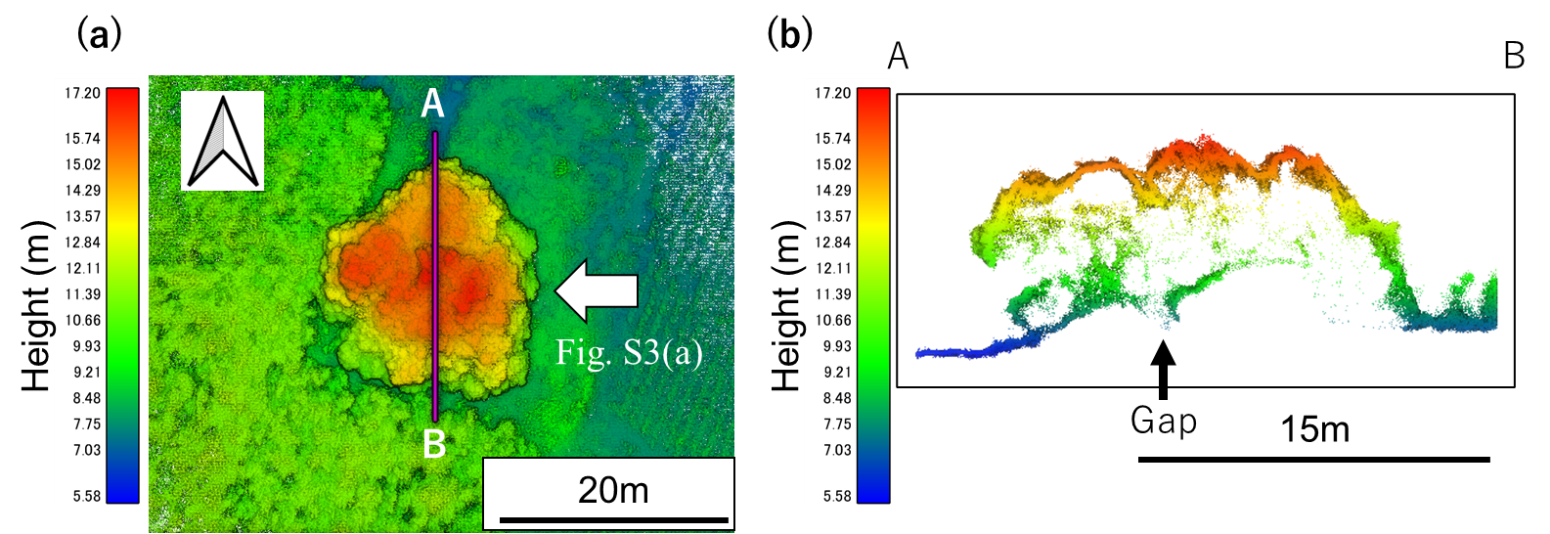


**Figure. S2**

(a) A height map using point cloud around the AS boulder. The white arrow shows the direction in which the Fig. S3(a) photo was taken. (b) A Cross section of a point cloud of the AS boulder. A and B represent each end of the transect as seen in Fig. S2a. There is a gap in the boulder, and it appears to be cracked. However, in reality, the bulge on the left side of the gap in (b) represents artificially piled stones while the right side represents the boulder.


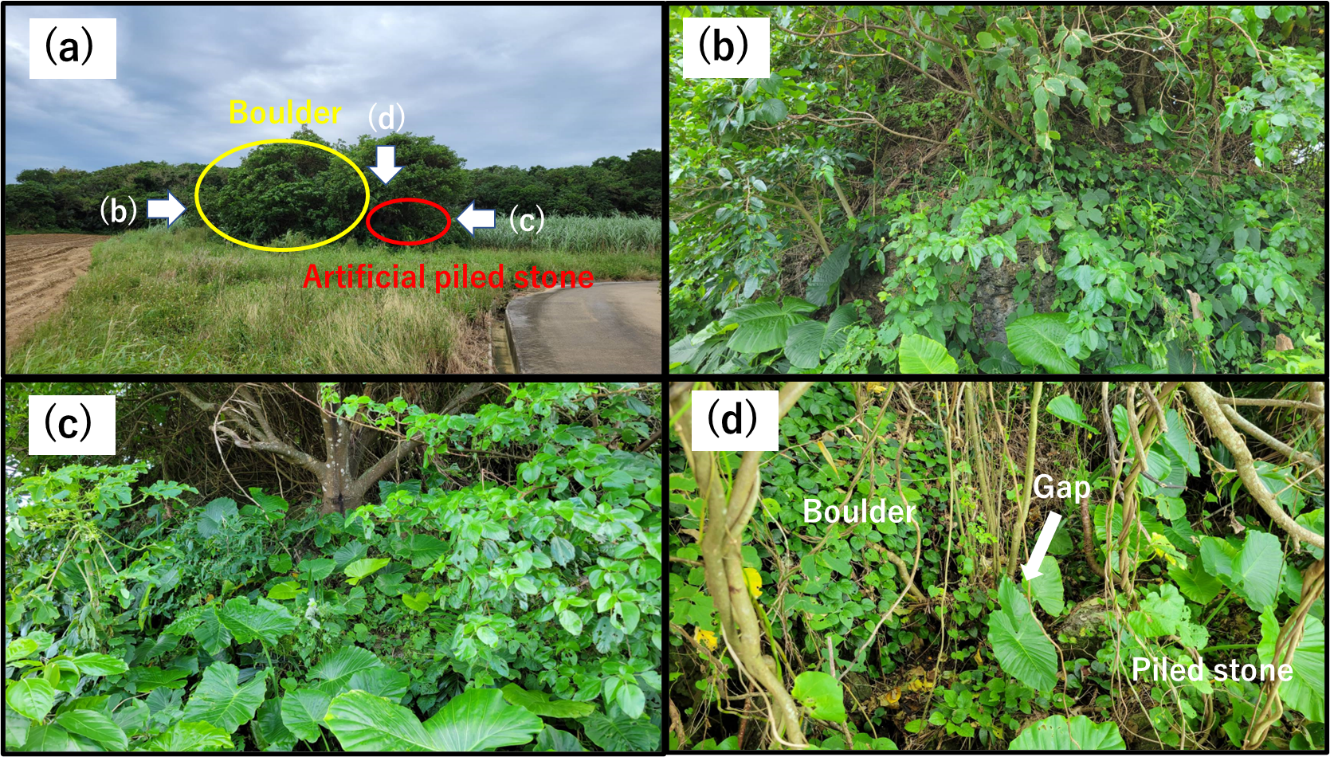


**Figure. S3**

(a) A photograph of the AS boulder taken from the direction of the white arrow in Fig. S2. The yellow areas show the boulder, while the red areas show the artificial piles of stones. Photographs (b)~(d) are taken from the direction of the white arrows shown in (a). (b) A photograph of the boulder’s surface. (c) A photograph of the surface of the piled stones. The surface is hard to see because it is covered by deep vegetation. (d) A photograph between the boulder and the piled stones. There is a gap between the two, as shown in Fig. S2(b).


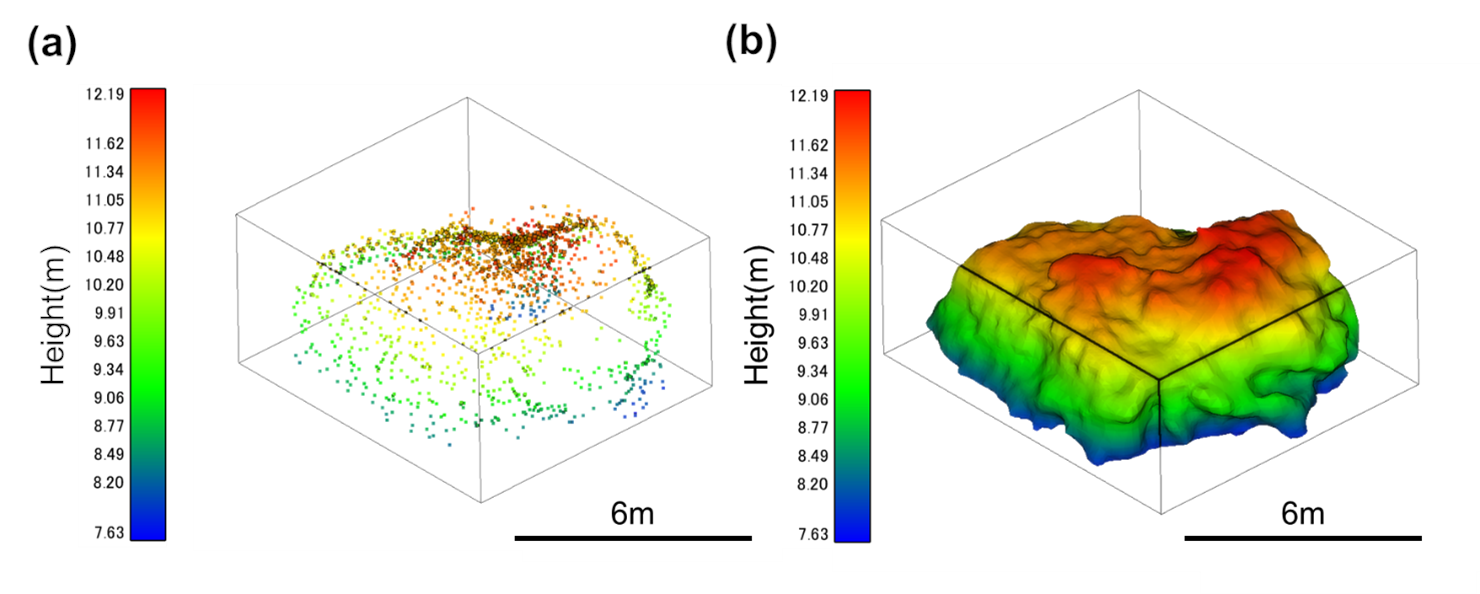


**Figure. S4**

(a) A point cloud data of the boulder after removing vegetation. (b) A 3D model of the AS boulder*.*

**Supplementary references**

1. Goto, K., Kawana, T. & Imamura, F. Historical and geological evidence of boulders deposited by tsunamis, southern Ryukyu Islands, Japan. *Earth-Science Reviews* **102**, 77-99 (2010).
2. Imamura, F., Goto, K. & Ohkubo, S. A numerical model for the transport of a boulder by tsunami. *Journal of Geophysical Research: Oceans* **113**, C01008 (2008).
